# Spatial histology and gene-expression representation and generative learning via online self-distillation contrastive learning

**DOI:** 10.1093/bib/bbaf317

**Published:** 2025-07-06

**Authors:** Qianyi Yan, Xuan Li, Jiangnan Cui, Jianming Rong, Jingsong Zhang, Pingting Gao, Yaochen Xu, Fufang Qiu, Chunman Zuo

**Affiliations:** School of Life Sciences, Sun Yat-sen University, No. 135, Xingang West Road, Haizhu District, Guangzhou 510275, Guangdong Province, China; College of Information Science and Technology, Donghua University, No. 2999, Renmin North Road, Songjiang District, Shanghai 201620, China; College of Computer Science and Technology, Jilin University, No. 2699, Qianjin Street, Changchun 130012, Jilin Province, China; College of Information Science and Technology, Donghua University, No. 2999, Renmin North Road, Songjiang District, Shanghai 201620, China; College of Information Science and Technology, Donghua University, No. 2999, Renmin North Road, Songjiang District, Shanghai 201620, China; Naval Healthcare Information Center, Faculty of Military Health Services, Naval Medical University, No. 800, Xiangyin Road, Pudong New District, Shanghai 200433, China; Shanghai Collaborative Innovation Center of Endoscopy, Endoscopy Center and Endoscopy Research Institute, Zhongshan Hospital, Fudan University, No. 180, Fuxing Road West, Xuhui District, Shanghai 200433, China; Shanghai Institute of Biochemistry and Cell Biology, Center for Excellence in Molecular Cell Science, Chinese Academy of Sciences, No. 320, Yueyang Road, Xuhui District, Shanghai 200031, China; Department of Neurosurgery, Huashan Hospital, Shanghai Medical College, Fudan University, National Center for Neruological Disorders, Shanghai Key Laboratory of Bran Function and Restoration and Neural Regeneration, Neurosurgical Institute of Fudan University, Shanghai Clinical Medical Center of Neurosurgery, No. 12, Wulumuqi Road West, Xuhui District, Shanghai 200032, China; School of Life Sciences, Sun Yat-sen University, No. 135, Xingang West Road, Haizhu District, Guangzhou 510275, Guangdong Province, China; Shanghai Collaborative Innovation Center of Endoscopy, Endoscopy Center and Endoscopy Research Institute, Zhongshan Hospital, Fudan University, No. 180, Fuxing Road West, Xuhui District, Shanghai 200433, China

**Keywords:** spatial transcriptomics data, multi-modal integration, histology-to-gene expression prediction, momentum-based contrastive learning

## Abstract

Spatial transcriptomics quantifies spatial molecular profiles alongside histology, enabling computational prediction of spatial gene expression distribution directly from whole slide images. Inspired by image-to-text alignment and generation, we introduce Magic, a self-training contrastive learning model designed for histology-to-gene expression prediction. Magic (i) employs contrastive learning to derive shared embeddings for histology and gene expression while utilizing a momentum-based module to generate pseudo-targets to reduce the impact of noise; and (ii) leverages a transformer-based decoder to predict the expression of 300 genes based on histological features. Trained on 75 760 spots from 56 breast cancer slices and validated on 11 026 spots from five independent slices, Magic outperforms existing methods in aligning and generating histology–gene expression data, achieving a 10% improvement over the second-best approach. Furthermore, Magic demonstrates robust generalization, effectively predicting gene expression in colorectal cancer samples and The Cancer Genome Atlas (TCGA) datasets through zero-shot learning. Notably, Magic’s predicted gene expression captures interpatient differences, highlighting its strong potential for clinical applications.

## Introduction

Deciphering the spatial enrichment and heterogeneity of gene expression within tissues is essential for uncovering the roles of genes in shaping tissue architecture and function [1]. However, conventional methods for quantifying gene expression, such as antibody-based histological staining or transcriptomics sequencing, are often labor-intensive, time-consuming, and costly, highlighting the urgent need for advanced and scalable computational solutions [2].

Spatially resolved transcriptomics (SRT) makes a significant breakthrough in preserving gene expression profiles within their spatial context, enabling a comprehensive characterization of tissue structure at unprecedented resolution [3–5]. By integrating histopathology images stained with hematoxylin and eosin (H&E), SRT technologies can precisely align histological features to specific spatial spots or cells, thus filling the gap between morphology and molecular biology. However, integrating these multimodal data from large-scale datasets is challenging due to sequencing and staining biases, inherent noise, and weak intermodality correlation.

Recently, computational methods have emerged to predict gene expression patterns directly from histological images. These approaches process image patches by transforming them into simplified representations, encoding them as feature vectors, and decoding them to reconstruct gene expression profiles, bridging image data with molecular insights. State-of-the-art methods utilize convolutional neural networks or transformers, such as Hist2RNA [6], Hist2ST [7], tRNAsformer [8], Transformer with Convolution and Graph-Node co-embedding method (TCGN) [9], BrST-Net [10], ST-Net [2], and HisToGene [11], while others adopt simpler linear encoders like Spatial Gene Expression Prediction from Local Graphs (SEPAL) [12] and HE2RNA [13]. To enhance embeddings, methods such as TCGN [9], SEPAL [12], the Integrated Graph and Image Deep Learning (IGI-DL) [14], and TRIPLEX [15] incorporate graph-based models, effectively capturing spatial relations within the data. Moreover, Bi-modaL Embedding for Expression Prediction (BLEEP) [16] employs contrastive learning to align histology with gene expression data by identifying similar reference profiles. Despite advancements, existing methods struggle to mitigate noise and weak correlations between histological images and gene expression data or to fully leverage highly variable genes within each spot, limiting their capacity to capture tissue heterogeneity and establish accurate links between histology and gene expression.

In this work, we propose Magic, an online self-supervised model designed to predict spatial gene expression from histological images. To address noisy and weakly correlated histology and gene expression datasets, Magic employs contrastive learning to derive shared embeddings while maintaining a momentum-based module to generate pseudo-targets for enhanced supervision [17]. Simultaneously, it utilizes a binary classification module to enforce consistent pairing of the multimodal representations. Subsequently, a transformer-based architecture is employed to deliver accurate gene expression predictions.

We trained Magic on 75 760 spots from 56 slices and validated it on 11 026 spots from five independent slices. Magic outperforms state-of-the-art methods in histology-gene expression alignment and generation, achieving a 10% improvement over the second-best method. Furthermore, the trained model successfully predicts colorectal cancer SRT and TCGA samples through zero-shot learning. Notably, Magic’s predicted gene expression effectively captured patient variations, highlighting its strong potential for clinical applications.

## Methods

### Magic model

**Figure 1:**
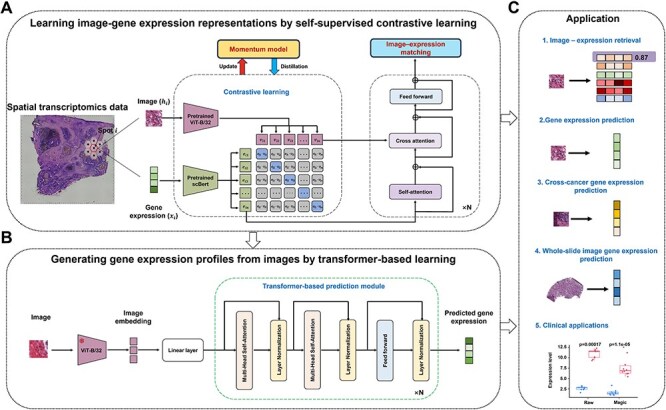
Overview of Magic. (A) Magic takes paired image and gene-expression data from each SRT spot as input, learning a shared image–gene expression representation space by integrating self-distillation contrastive learning with an image–gene expression classification model. (B) Magic utilizes a pretrained image encoder from step a to extract histological features, which are then used by a transformer-based decoder to predict gene expression. (C) Magic enables a variety of downstream applications, including image-expression retrieval and prediction, cross-cancer prediction via zero-shot learning, and potential clinical implementations.

Magic enables histology and gene-expression alignment and generation by integrating histological images ($H=({h}_1,\dots, {h}_n),{h}_i\in{R}^{32\times 32}$) and gene expression profiles ($X=({x}_1,\dots, {x}_n),{x}_i\in{R}^{1\times 2000}$) from SRT data, where $n$ indicates the number of spots (Fig. 1A–C). This integration is achieved through a two-step approach. Specifically, (i) Magic extracts histological features ($V= ({v}_1,\dots, {v}_n),{v}_i\in{R}^{1\times 512}$) and gene expression features ($E=\left({e}_1,\dots, {e}_n\right),{e}_i\in{R}^{1\times 512}$) from the histological images and gene expression data using a ViT-B/32 encoder and an scBert-based encoder, respectively. The extracted features are projected into a shared embedding space through contrastive learning [18], guided by a momentum distillation mechanism and supervised by a binary classification objective (IGM module), ensuring robust cross-modal representation (Fig. 1A); and (ii) a transformer-based decoder utilizes histological features extracted by the ViT-B/32 encoder from stage (1) to predict raw 300-dimensional gene expression profiles, simultaneously capturing biological signals and batch effects (Fig. 1B).

### Learning image–gene expression representations by self-distillation contrastive learning

Motivated by ViT’s exceptional capability to extract meaningful image features [19], Magic employs ViT-B/32 as the histology encoder ($H\left(\bullet \right)$) to capture histological features ($V$). To fully harness the information in gene expression data, 2000 highly variable genes were selected for each spot. The scBert mode pretrained on large-scale single-cell RNA-seq data [20] is combined with a convolutional layer and a global average pooling (GAP) layer as the encoder ($G\left(\bullet \right)$) to learn gene expression features ($E$) (Supplementary Fig. S1). Specifically, Magic uses the scBert model to project gene expression data ${x}_i$into a low-dimensional feature space, where the scBert model consists of 12 transformer layers with eight attention heads. Subsequently, a 2D convolution layer [21] is applied with a kernel size of (7,5) and padding of (0,2) to further extract local features. Next, a GAP layer is used to compute the mean across all channels, reducing dimensionality and enhancing global context awareness. The resulting feature map is then passed through a fully connected network with three layers, followed by a Rectified Linear Units (ReLU) [22] activation function to introduce nonlinearity. To mitigate overfitting, a Dropout layer with a rate of 0.5 is applied after the final fully connected layer. The output of this process is the final gene expression embedding ${e}_i$.


$$ {h}_i= scBert\left({x}_i\right) $$

$$ {h}_i^{\prime }= Conv2\left({h}_i\right) $$

(1) \begin{equation*} {h}_i^{\prime \prime }=\frac{1}{L}\sum_{l=1}^L{h}_{i,l}^{\prime } \end{equation*}

$$ {z}_i= ReLU\left(W{h}_i^{\prime \prime }+b\right) $$

$$ {e}_i= Dropout\left({z}_i\right) $$

where ${h}_i$ represents the gene expression embedding extracted by the scBert model; ${h}_i^{\prime }$ denotes the feature maps output by the convolutional layer; ${h}_i^{\prime \prime }$ is the feature vector processed by the GAP layer; $W$ and $b$ represent the weight matrix and bias term of the fully connected layer, respectively; $L$ indicates the number of feature vectors by GAP layer; and other operators are defined in Supplementary Table S6.

To address noise in histology and gene expression data, we leveraged the momentum encoder $M\left(\bullet \right)$ to guide the training of the two encoders. The core idea of $M\left(\bullet \right)$ is to serve as a teacher, maintaining stable representations to counteract instability caused by sample diversity or randomness during training. Specifically, for each paired histology ${h}_i$ and gene expression ${x}_i$ for spot $i$, we simultaneously extracted representations using the primary encoder $\left(H (\bullet ),G\left(\bullet \right)\right)$ and their counterparts in the momentum model $\left({M}_H (\bullet ),{M}_G\left(\bullet \right)\right)$.


(2) \begin{equation*} {v}_i=H\left({h}_i\right),{v}_i^M={M}_H\left({h}_i\right) \end{equation*}

$$ {e}_i=G\left({x}_i\right),{e}_i^M={M}_G\left({x}_i\right) $$

where $H\left({h}_i\right)$ and ${M}_H\left({h}_i\right)$ represent the histological representation learned from the image encoder and its momentum counterpart, respectively, while $G\left({x}_i\right)$ and ${M}_G\left({x}_i\right)$ indicate the gene-expression representation learned from the gene-expression encoder and its momentum counterpart.

In momentum distillation, to maintain consistency between the primary encoder and the momentum encoder during training, the parameters of the momentum encoder (${\theta}_m$) are updated gradually from the parameters of the primary encoder ($\theta$) at a controlled slow rate:


(3) \begin{equation*} {\theta}_m={\beta \theta}_m+\left(1-\beta \right)\theta \end{equation*}

where $\beta$ is the momentum coefficient that determines the update speed of the momentum encoder, with a default value of 0.995. The momentum distillation mechanism provides guidance for the primary encoder to progressively align with the momentum encoder, ensuring the stability of encoding training.

Moreover, we applied contrastive learning to establish relationships between histology and gene expression in the representation space while transferring knowledge from the momentum encoder to the primary encoder. This approach strengthens the similarity between paired images and gene expression representations while minimizing the similarity of unpaired ones. The histology–gene expression contrastive loss is defined as the cross-entropy ($CE$) between $p$ and $y$:


(4) \begin{align*} {L}_{con}&=\frac{1}{2}{E}_{\left(H,X\right)\sim D}\left[ CE\left({y}^{h2g}(H),{p}^{h2g}(H)\right)+ CE\left({y}^{g2h}(X),{p}^{g2h}(X)\right)\right]\nonumber\\{p_k}^{h2g}(V)&=\frac{\exp \left( sim\left(V,{e}_k\right)/\tau \right)}{\sum_{s=1}^S\exp \left( sim\left(V,{e}_s\right)/\tau \right)}\nonumber\\{p_k}^{g2h}(E)&=\frac{\exp \left( sim\left(E,{v}_k\right)/\tau \right)}{\sum_{s=1}^S\exp \left( sim\left(E,{v}_s\right)/\tau \right)}\end{align*}

where $\tau$ indicates the temperature hyperparameter, set to a default value of 0.07; $S$ is the number of spots per batch; $V$ and $E$ denote the histological and gene expression feature matrices for each batch; and ${y}^{h2g}$ and ${y}^{g2h}$ represent the ground truth, computed as follows:


(5) \begin{align*} {y}^{h2g}&=\alpha \bullet{p_k}_M^{h2g}\left({V}^M\right)+\left(1-\alpha \right)\bullet A \nonumber\\{y}^{g2h}&=\alpha \bullet{p_k}_M^{g2h}\left({E}^M\right)+\left(1-\alpha \right)\bullet A\nonumber\\{p_k}_M^{h2g}\left({V}^M\right)&=\frac{\exp \left( sim\left({V}^M,{e}_k^M\right)/\tau \right)}{\sum_{s=1}^S\exp \left( sim\left({V}^M,{e}_s^M\right)/\tau \right)}\nonumber\\{p_k}_M^{g2h}\left({E}^M\right)&=\frac{\exp \left( sim\left({E}^M,{v}_k^M\right)/\tau \right)}{\sum_{s=1}^S\exp \left( sim\left({E}^M,{v}_s^M\right)/\tau \right)}\end{align*}

where $A$ is a diagonal matrix with diagonal elements set to 1 to indicate positive pairs and the off-diagonal elements set to 0 to represent negative pairs. The parameter $\alpha$ controls the relative weight between the two components, with a default value of 0.4. Notably, the matrix ${p}_M$ represents the similarity scores between histological and gene expression features obtained from the momentum encoder, serving as an additional supervised signal to enhance the training of the primary encoder.

In addition, we proposed a binary classification model to achieve fine-grained alignment between histology and gene expression representations. Specifically, we leveraged the self-attention mechanism to enhance gene expression features, producing ${E}_{self}$. This was followed by a cross-attention mechanism to integrate ${E}_{self}$ with histological features $V$, generating joint features ${Z}_{cross}$, which were used as the Query, while the histological representations served as both the Key and Value. The feedforward network ($FFN$) [23], incorporating a residual structure that combines the input with the output and applies LayerNorm [24] for standardization, is designed to learn multi-modal representation $Z$, computed as follows:


(6) \begin{align*} Z&= LayerNorm\left({Z}_{cross}+ FFN\left({Z}_{cross}\right)\right) \nonumber\\ FFN\left({Z}_{cross}\right)&={W}_2\left( ReLU\left({Z}_{cross}{W}_1+{b}_1\right)\right)+{b}_2\nonumber\\{Z}_{cross}&= LayerNorm\left({Z}_{self}+ CrossAttention\left({Q}_{Z_{cross}},{K}_H,{V}_H\right)\right)\nonumber\\ CrossAttention &\left({Q}_{Z_{cross}},{K}_H,{V}_H\right)= Softmax\left(\frac{Q_{Z_{cross}}{K}_H^T}{\sqrt{d_k}}\right){V}_H\nonumber\\{Q}_{Z_{cross}}&={Z}_{self}{W}_Q,{K}_H={V}_i{W}_K,{V}_H={V}_i{W}_V \nonumber\\{Z}_{self}&= LayerNorm\left({x}_i+ Attention\left(Q,K,V\right)\right) \end{align*}

where ${W}_1$, ${W}_2$, ${b}_1$, and ${b}_2$ indicate the learnable parameters of the model. ${W}_Q$, ${W}_K$, and ${W}_V$ are all learnable parameter matrices. $Q$, $K$, and $V$ represent Query, Key, and Value, respectively. We further employed the image and gene expression matching model to utilize multi-modal features $Z$ for label prediction, where each image–gene pair is designed as a positive sample, while the image representation most similar to the gene-expression representation, as determined by both encoders from the previous training round, is selected as its hardest negative sample. The loss function is summarized as follows:


(7) \begin{equation*} {L}_{IGM}=-\sum{y}_i\log \left({p}_i\right)+\left(1-{y}_i\right)\log \left(1-{p}_i\right) \end{equation*}

where ${y}_i$ and ${p}_i$ represent the label vector of each pair of histology–gene expression from the ground truth and the prediction of IGM module.

### Image-to-gene expression data generative learning

To accurately predict the corresponding gene expression from histological images, we leveraged a transformer-based decoder to predict the gene expression of 300 genes (Fig. 1B). We utilized the frozen image encoder $I\left(\bullet \right)$ to extract image representations $V$, which were progressively mapped through linear layers to a high-dimensional space, producing the output feature ${E}^{\prime }$. The transformer decoder, comprising stacked layers of multi-head self-attention, normalization layer, and a feedforward network, extends the self-attention mechanism with multiple parallel attention heads, whose outputs are concatenated for enhanced representation.


(8) \begin{align*} MultiHead\left(Q,K,V\right)&={W}_o\left( Concat\left({head}_1\dots, {head}_h\right)\right)\nonumber\\{head}_i&= Attention\left({Q}_i,{K}_i,{V}_i\right) \end{align*}

where ${Q}_i$, ${K}_i$, and ${V}_i$ denote the $i$th input Query, Key, and Value matrices, respectively; ${head}_i$ represents the output of the $i$th attention head; and ${W}_o$ is a learnable linear projection matrix. After each multi-head attention mechanism, a feedforward neural network is applied to further refine and process the features. Layer normalization is used after both the multi-head attention mechanism and the feedforward network, complemented by residual connections. The feature vector ${E}^{\prime }$ is passed through the transformer, which produces the predicted gene expression ${X}_p$ for the image. This prediction is then compared with the raw gene expression $X$, and the loss is calculated using the mean squared error (MSE), defined as:


(9) \begin{equation*} {L}_{MSE}={\left\Vert X-{X}_p\right\Vert}_2 \end{equation*}

The comparison results confirm that the selected hyperparameters—momentum, learning rate, and weight decay—enable efficient convergence and optimal overall performance (Supplementary Fig. S7 and Supplementary Table S5). Furthermore, as demonstrated in Supplementary Fig. S8, both training and validation losses converge and stabilize within 50 epochs, providing evidence that the current dataset facilitates effective model optimization.

### Datasets and preprocessing

#### Spatially resolved transcriptomics and TCGA samples

The human SRT and bulk datasets used in this study were obtained from the 10x Genomics website [25] and published studies [26, 33] (Supplementary Table S1). These datasets included 48 triple-negative breast cancer samples, 3 invasive ductal carcinoma samples, 3 invasive lobular carcinoma samples, 5 colorectal cancer samples, and 25 breast cancer samples from the TCGA database. Each SRT dataset contains histology, gene expression data, and spatial location, while each TCGA sample includes WSI and gene expression data.

#### Preprocessing

For each SRT sample, spot images were center-cropped to a size of $W\times H$ ($40\times 40$ pixels) [3] based on our comparative experiments (Supplementary Fig. S9). The cropped histological images were then resized to a fixed resolution compatible with the ViT-B/32 encoder. During histology–gene expression representation learning, the top 2000 highly variable expressed genes were selected as input for each spot’s gene expression vector. Gene expression values at each spot were normalized by dividing each gene’s Unique Molecular Identifier (UMI) count by the total UMI count across all genes in that spot, multiplying by 1 000 000, and then applying a natural logarithmic transformation. For gene expression prediction from histology, the top 300 highest expression genes across all trained spots were selected as the target for generation. Additionally, histological images were preprocessed using cropping, random flipping, random brightness, and contrast adjustments.

Moreover, to evaluate whether our predictions can capture sample diversity, we randomly selected 10 samples from the normal and stage IV groups of the TCGA breast cancer dataset. A strategy was employed to select 20 histological images ($512\times 512$ pixels) per WSI that best represent biological variations, based on the following quantification metrics:


(10) \begin{equation*} {N}_t\bullet \tanh \left({T}_t\right) \end{equation*}

where ${N}_t$ and ${T}_t$ indicate the percentage of nuclei and tissue in the image region $t$, with patches containing <60% tissue content being excluded.

### Evaluation of image and gene expression representation alignment accuracy

To assess if or not paired image and gene expression for each spot are better aligned in the representation space compared to its spatially adjacent spots, we defined a metric based on the cosine similarity between image and gene expression, calculated as follows:


(11) \begin{equation*} Accuracy=\frac{N_{correct}}{N} \end{equation*}

where ${N}_{correct}$ indicates the number of paired image and gene expression profiles with the highest cosine similarity, and $N$ denotes the total number of image and gene-expression pairs tested.

### Assessment of gene expression prediction accuracy

We leveraged gene- and spot-level Pearson correlations to assess the accuracy of computational models in predicting gene expression from histopathological images, calculated as follows:


(12) \begin{equation*} PCC=\frac{\sum_{i=1}^n\left({x}_i-\overline{x}\right)\left({x}_{pi}-\overline{x_p}\right)}{\sqrt{\sum_{i=1}^n{\left({x}_i-\overline{x}\right)}^2{\sum}_{i=1}^n{\left({x}_{pi}-\overline{x_p}\right)}^2}} \end{equation*}

where ${x}_i$ and ${x}_{pi}$ represent the true and predicted gene expression value for gene $i$ across spots (or sample $i$ across 300 genes), while $\overline{x}$ and $\overline{x_p}$ denote the mean of ${x}_i$ and ${x}_{pi}$ across $n$ spots (or genes). Additionally, for the TCGA dataset, we utilized MSE and MAE to evaluate the prediction performance:


(13) \begin{align*} MSE&={\left\Vert X-{X}_p\right\Vert}_2 \nonumber\\ MAE&={\left\Vert X-{X}_p\right\Vert}_1 \end{align*}

## Results

### Overview of Magic

Magic facilitates the alignment and generation of histology and gene expression from SRT data through self-training multimodal integration (Fig. 1A–C). To enhance the association from paired noisy multimodal data, a momentum distillation mechanism generates pseudo-targets to guide contrastive learning between image and gene expression representations, while a binary classification model ensures fine-grained alignment between the two representations. Subsequently, a transformer-based model is utilized to predict gene expression profiles from image representations.

Magic employs the ViT-B/32 and scBert models to transform histological images (${h}_i$) and gene expression (${x}_i$) from the spot $i$, extracting histological features (${v}_i$) and gene expression features (${e}_i$), respectively. It utilizes contrastive learning to establish associations between these two feature spaces. Additionally, Magic integrates a momentum-based model as an evolving teacher to generate pseudo-targets for feature pairs, providing supplementary supervision for the contrastive learning process. Moreover, Magic incorporates a cross-attention-based multimodal fusion model to effectively learn multimodal features and utilizes an image-gene expression matching (IGM) module to achieve fine-grained alignment between paired representations (Fig. 1A and Supplementary Fig. S1). Subsequently, Magic leverages a transformer-based model to map learned histological features to generate gene expression profiles (Fig. 1B).

To emphasize the advantages of Magic, we designed several comparative experiments: (i) using shared representation space learned via contrastive learning between ViT-B/16 and gene expression, compared to ViT-B/32, to demonstrate the superior efficiency of ViT-B/32; (ii) integrating contrastive learning with a momentum model to enhance representation learning under noisy conditions; (iii) incorporating multimodal fusion with a binary classifier for histology–gene expression matching, showing the benefit of hard negatives in improving discriminative power; and (iv) directly predicting gene expression from histological features extracted by a pretrained ViT-B/32 encoder using the same transformer decoder as Magic (referred to as ViT-naïve), to assess the importance of histology–gene expression alignment module.

### Magic facilitates histology and gene expression alignment

**Figure 2:**
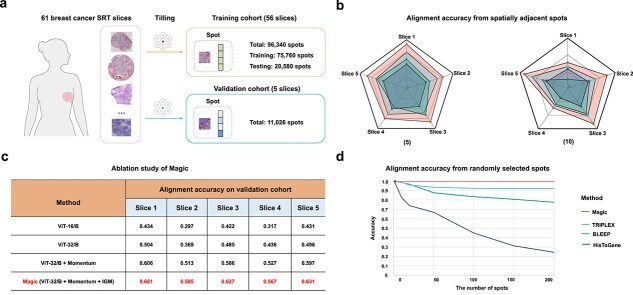
Magic enables image–gene expression matching. (A) Description of 61 breast cancer SRT slices used for model training, testing, and validation. A total of 75 760 points (70%) from 56 slices were used for model training, while the remaining 20 580 points (30%) were reserved for testing. Additionally, five independent SRT slices were used as a separate validation dataset. Selected images were adapted from Servier Medical Art, under the Creative Commons Attribution 4.0 International License. (B) Radar plots showing image–gene expression alignment accuracy for spatially adjacent 5 spots (left panel) and 10 (right panel) spots on five validation SRT slices. (C) Table comparing the performance of different components of Magic in image–gene expression alignment accuracy for spatially adjacent five spots on five validation SRT slices. (D) Line plot illustrating alignment accuracy by HisToGene, BLEEP, TRIPLEX, and Magic in identifying gene expression from varying numbers of randomly selected spots, based on cosine similarity between image and gene-expression embeddings.

To demonstrate the efficiency of Magic in aligning histology and gene expression in the low-dimensional features, we manually collected 61 SRT sections comprising a total of 96 340 spots from previous studies [26]. Among the 56 slices, 75 760 spots were used for model training, and the remaining 20 580 spots were reserved for testing. Additionally, five independent SRT slices (11 026 spots) were used as separate datasets to further validate the model’s generalization ability (Fig. 2A and Supplementary Table S1). We benchmarked Magic against three recently published methods such as HisToGene, BLEEP, and TRIPLEX. The same datasets used for Magic were employed to train and test these models for a fair comparison. As neighboring spots often share similar expression patterns, alignment accuracy was defined as identifying paired gene expression from 5 or 10 spatially adjacent spots using cosine similarity between image and gene expression embeddings to evaluate different methods.

By comparison, we found that (i) Magic outperforms other methods in accurately aligning image and gene-expression embeddings on the five validation SRT slices, achieving a 10% improvement over the second-best method, TRIPLEX, while HisToGene exhibits the lowest performance (Fig. 2B and Supplementary Table S2); (ii) Magic significantly outperforms all other approaches (*P* = 7.937e-03) (Supplementary Fig. S2); (iii) The alignment accuracy improved by 7.8%, 10.8%, and 5.2% for ViT-B/32, ViT-B/32 + momentum, and Magic (i.e. ViT-B/32 + momentum + IGM), respectively, compared to ViT-B/16, ViT-B/32, and ViT-B/32 + momentum, highlighting that the necessity of each Magic component and the critical role of momentum in enhancing model performance (Fig. 2C); and (iv) Magic consistently achieves high matching accuracy across varying numbers of randomly selected spots, demonstrating its efficiency in capturing the biological specificity of image and gene expression embeddings (Fig. 2D).

Taken together, Magic excels in aligning image and gene expression embeddings with high accuracy.

### Magic enables gene expression prediction from image

To further evaluate the performance of Magic in predicting gene expression from histology (Fig. 1B), we trained a transformer-based model to predict 300 highly expressed common genes using 56 training slices (Fig. 2A). For comparison, the same datasets were used to train ST-Net, HisToGene, BLEEP, and TRIPLEX. The efficiency of these prediction models was assessed by calculating Pearson correlation at both spot and gene levels between raw and predicted data within five validation slices.

**Figure 3:**
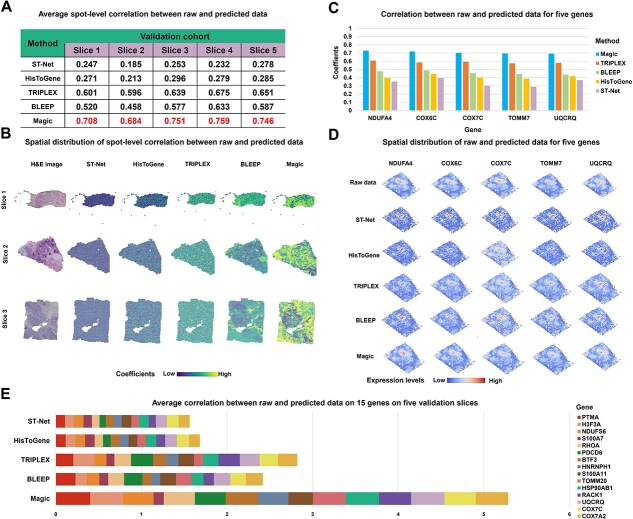
Magic facilitates predicting gene expression from histology. (A) Table summarizing the mean spot-level correlation between raw and predicted gene expression achieved by Magic, TRIPLEX, BLEEP, HisToGene, and ST-Net, on five validation SRT slices. (B) Spatial H&E images and distributions of correlation values between raw and predicted gene expression by ST-Net, HisToGene, BLEEP, TRIPLEX, and Magic, on three validation SRT slices. (C) Bar plots showing correlation values between raw and predicted gene expression for five highly correlated genes (*NDUFA4*, *COX6C*, *COX7C*, *TOMM7*, and *UQCRQ*) achieved by Magic, TRIPLEX, BLEEP, HisToGene, and ST-Net, on one validation slice: GSM6433625_398B. (D) Spatial distributions of raw and predicted gene expression for five genes (*NDUFA4*, *COX6C*, *COX7C*, *TOMM7*, and *UQCRQ*) by ST-Net, HisToGene, BLEEP, TRIPLEX, and Magic on one validation slice: GSM6433625_398B. (E) Bar plots displaying the mean gene-level correlation for 15 genes across five validation slices between raw and predicted gene expression achieved by ST-Net, HisToGene, BLEEP, TRIPLEX, and Magic, with each color representing one gene.

In summary, we found that (i) Magic outperforms all other methods in spot-level correlations between raw and predicted data, achieving a mean improvement of 10% compared to the second-best method, TRIPLEX, while ST-Net shows the lowest performance (Fig. 3A and B). Additionally, Magic significantly performs better than other methods (*P* = 2.857e-02) (Supplementary Fig. S3); and (ii) Magic also achieves the highest gene-level correlations for five genes (*NDUFA4*, *COX6C*, *COX7C*, *TOMM7*, and *UQCRQ*), with a similar mean improvement of 10% over TRIPLEX, and ST-Net again performing the worst. Notably, *NDUFA4* is associated with immune modulation, *PD-L1* expression, and breast cancer prognosis [27], while altered expression of *COX6C* and *COX7C*, subunits of cytochrome c oxidase, is linked to poor cancer prognosis [28]. *TOMM7* is involved in mitochondrial protein import and may contribute to therapy resistance in breast cancer. *UQCRQ*, a subunit of the mitochondrial respiratory chain, plays a role in oxidative phosphorylation and has been linked to poor treatment response in breast cancer [29]. Furthermore, the spatial distributions of these five genes predicted by Magic exhibit superior alignment with raw data compared to other methods, underscoring Magic’s robustness in preserving spatial patterns (Fig. 3C and D); (iii) Magic demonstrates greater stability and robustness in gene-level correlations for 10 randomly selected genes, consistently outperforming other four methods across five validation slices (Fig. 3E); and (iv) Magic consistently outperforms ViT-naive in gene expression prediction, demonstrating that the histology–gene expression alignment module plays a key role in bridging the modality gap and contributes predictive power beyond that of a standalone transformer decoder (Supplementary Fig. S4).

In short, Magic proves to be a highly effective tool for predicting gene expression from histological images, driven by its precise alignment of multimodal features in a shared low-dimensional space and the advanced design of its decoder architecture.

### Magic enables cross-cancer prediction through zero-shot transfer learning

We hypothesized that consistent patterns exist between tumor histology and gene expression across different tissue types and that Magic could effectively capture these relationships. Specifically, we observed that ~250 out of 300 genes expressed in colorectal cancer also appear in the breast cancer dataset used for training (Supplementary Fig. S5). To test this, we evaluated Magic’s performance in predicting gene expression for colorectal cancer SRT datasets using a model trained exclusively on breast cancer SRT data, without any fine-tuning—a capability referred to as zero-shot learning [30]. For comparison, we also assessed the performance of ST-Net, HisToGene, TRIPLEX, and BLEEP, which were trained on the same datasets, to evaluate their ability to align histology with gene expression and predict gene expression from histological features.

**Figure 4:**
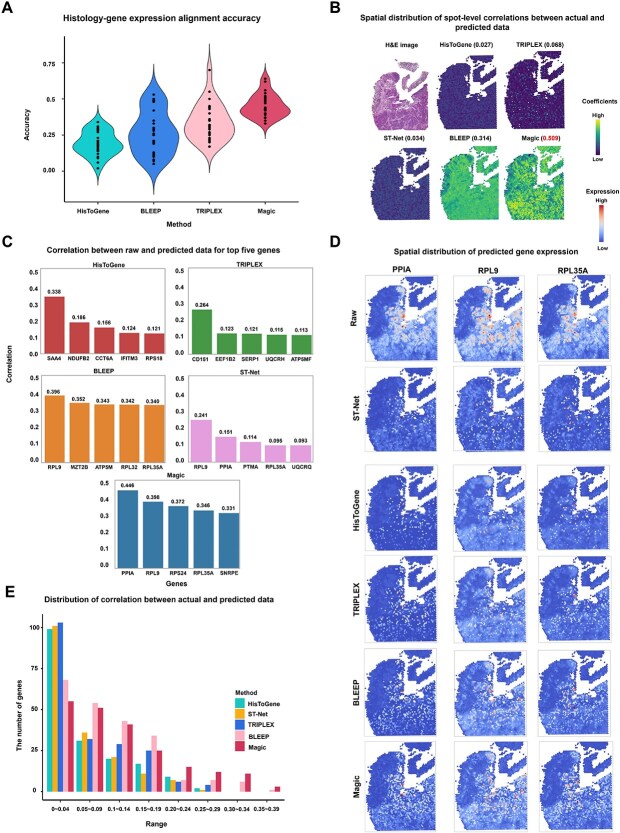
Magic achieves cross-cancer image–gene expression alignment and prediction through zero-shot learning. (A) Boxplot illustrating the alignment accuracy between image and gene expression representations for HisToGene, BLEEP, TRIPLEX, and Magic, based on cosine similarity between a spot image and 14 randomly selected spot gene expressions. This evaluation was performed on five different colorectal cancer tissue slices, repeated 100 times per experiment, across five experiments. For each boxplot, the center line, box limits, and whiskers separately indicate the median, upper and lower quartiles, and 1.5× interquartile range. (B) Spatial H&E image and distributions of spot-level correlation values between raw and predicted gene expression for ST-Net, HisToGene, BLEEP, TRIPLEX, and Magic, on one colorectal cancer tissue slice: ST-colon1. (C) Bar plot comparing the correlation values of five highly correlated genes between raw and predicted gene expression by ST-Net, HisToGene, BLEEP, TRIPLEX, and Magic on one colorectal cancer tissue slice: ST-colon1. (D) Spatial distributions of raw and predicted gene expression for three genes (*PPIA*, *RPL9*, and *RPL35A*) by ST-Net, HisToGene, BLEEP, TRIPLEX, and Magic on one validation slice: ST-colon1. (E) Histogram depicting the distribution of positive correlation values between raw and predicted gene expression by ST-Net, HisToGene, BLEEP, TRIPLEX, and Magic, showing the number of genes across different correlation ranges.

In summary, we observed that (i) for HisToGene, TRIPLEX, and Magic, the alignment accuracy between histology and gene expression in colorectal cancer is over 10% lower than in breast cancer, while BLEEP shows a smaller drop of 9%. Despite this performance gap, Magic still outperforms the second-best method, BLEEP, by 11%. (Fig. 4A and Supplementary Tables S2 and S3). The relatively poor generalization of HisToGene and TRIPLEX under zero-shot settings may stem from their inability to handle intercancer variability. HisToGene lacks modality alignment, while TRIPLEX relies solely on supervised fusion, failing to build a modality-invariant space and is thus sensitive to domain shifts; and (ii) although Magic’s spot-level correlation between predicted and ground truth gene expression drops by ~20% in colorectal cancer compared to breast cancer, it still surpasses all other methods. In contrast, predictions from ST-Net, HisToGene, and TRIPLEX exhibit little to no meaningful similarity to the real data (Fig. 4B). These results indicated that the model’s reliance on surrounding niches limits its ability to perform cross-cancer predictions, as cancer samples exhibit distinct cellular niches; (iii) for the top five highly correlated genes, the predictions by Magic demonstrate higher correlation compared to those from other methods, with the spatial distribution of the prediction aligning more consistently with real data (Fig. 4C and D); and (iv) Magic identifies a greater number of genes with correlation values higher than 0.2 between raw and predicted data, highlighting its ability to effectively capture sample specificity (Fig. 4E).

Magic enables image and gene-expression alignment and generation, even under the challenging conditions of colorectal cancer datasets, without requiring fine-tuning. This demonstrates its ability to capture tumor-type-independent relationships between image and gene expression representations.

### Magic reveals biological variations across TCGA samples

**Figure 5:**
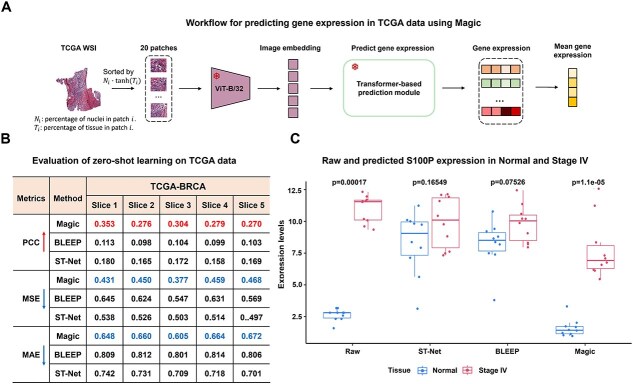
The application of Magic on TCGA datasets. (A) Workflow for gene expression prediction from whole slide image (WSI) on TCGA data using Magic. For each WSI, 20 images with dimensions of 512 × 512 pixels were selected. The pretrained ViT-B/32 encoder in Magic was used to extract histological features from each image. Gene expression was then predicted using Magic’s pretrained transformer-based decoder. The mean of the 20 predicted gene expression values was taken as the final gene expression prediction for the WSI. (B) Table displaying the gene-level correlation, MSE, and MAE between actual and predicted gene expression data for ST-Net, BLEEP, and Magic across five WSIs. (C) Boxplot showing the distribution of raw predicted *S100P* gene expression in 10 randomly selected samples from normal and stage IV groups in the TCGA BRCA dataset. A Wilcoxon test was performed to compare differences between normal and stage IV samples. For each boxplot, the center line, box limits, and whiskers separately indicate the median, upper and lower quartiles, and 1.5× interquartile range.

A key strength of Magic is its ability to predict gene expression from histological images without relying on spatial location information. To assess its versatility and clinical potential, we applied Magic to breast cancer samples from the TCGA dataset, testing its robustness under challenging conditions [31]. The evaluation performance on TCGA is affected by several key differences between the TCGA and SRT datasets: (i) TCGA data are derived from bulk genomic sequencing of whole tumors or normal tissues and lack spatial localization information; (ii) substantial differences in the histological image characteristics caused by variations in staining protocols across datasets. However, Magic demonstrates remarkable robustness and efficiency in predicting gene expression from histological images in the TCGA dataset. Sparse tissue areas were excluded from the whole slide image (WSI), and denser regions were divided into 20 grid-based sections, with the average predicted gene expression across these regions used to represent expression levels for each sample (Fig. 5A). For comparison, we utilized ViT-naive, ST-Net, and BLEEP, which do not rely on spatial location, to predict gene expression.

Our analysis revealed that Magic outperforms ViT-naive, ST-Net, and BLEEP in gene expression prediction, achieving ~20% improvement in tissue-level correlation, mean square error, and mean absolute error between raw and predicted data (Fig. 5B). Notably, among the 300 genes analyzed, *S100P*, a key gene implicated in breast cancer metastasis [32], shows significant differential expression between stage IV tumors and normal tissues. To evaluate Magic’s ability to capture biological variation, we analyzed 10 samples each from stage IV tumor and normal groups. Magic’s predictions exhibit a significant difference in gene expression between the two groups, with a lower $P$-$value$ than that observed in raw data. In contrast, ViT-naive, ST-Net, and BLEEP fail to demonstrate a significant difference between raw and predicted data (Fig. 5C and Supplementary Fig. S6).

Magic effectively captures sample-specific characteristics and biological variations, even in zero-shot learning scenarios, highlighting its transformative in clinical applications.

## Discussion

In this study, we proposed Magic, a self-training contrastive learning model for spatial histology and gene-expression alignment and generation from SRT datasets. To address the challenges of noisy and weakly correlated multimodal data, Magic leverages a momentum distillation mechanism to generate pseudo-targets for contrastive learning and employs a binary classification model to enforce consistent pairing, enabling fine-grained alignment between histological and gene expression representations. Furthermore, a transformer-based decoder utilizes a multi-head self-attention mechanism to capture long-range dependencies within histological representations, effectively predicting the expression levels of highly expressed genes. Ablation studies validated the importance and contributions of Magic’s key components, underscoring its robust design and performance (Fig. 2C).

We benchmarked Magic against three state-of-the-art methods for histology–gene expression alignment and generation on previously unseen datasets, including independent breast cancer validation cohorts, colorectal cancer samples, and TCGA datasets, to assess its versatility and generalizability. Magic demonstrated a remarkable ability to capture tumor-type-independent relationships between histological images and gene expression representations. Additionally, its predicted gene expression accurately reflected sample-specific characteristics and biological variations, underscoring its significant potential for clinical application.

In future studies, we aim to enhance the current model in several key directions: (i) with the growing availability of single-cell resolution spatial transcriptomics data, we plan to develop a more advanced model capable of predicting gene expression at finer spatial and cellular resolutions; (ii) to address challenges associated with noisy and low-quality histology and gene expression data, we will explore data augmentation techniques and leverage known gene–gene relationships to improve robustness and accuracy; (iii) to enhance the model’s generalizability and performance, we plan to expand training on diverse tissue datasets, thereby enabling broader research and clinical applications; and (iv) although Magic requires more training time, memory consumption, and inference time compared to HisToGene and BLEEP, its computational resource demands remain lower than those of TRIPLEX (Supplementary Table S4). We recognized this limitation and will focus on optimizing the model’s efficiency in future studies; and (v) while our results suggest an enhanced biological signal and reduced technical noise, we acknowledged that future work is needed to design more sophisticated algorithms that systematically disentangle and balance biological variation and batch effects.

**Key Points**

- Magic employs contrastive learning to unify histology and gene expression into shared embeddings. A momentum-based model generates pseudo-targets to reduce noise, while a transformer-based decoder predicts the expression of 300 genes from histological features.
- Magic surpasses existing methods in aligning and generating histology–gene expression data, achieving a 10% improvement over the second-best approach.
- Magic exhibits robust generalization, effectively predicting gene expression in colorectal cancer and TCGA datasets through zero-shot learning. Its predictions capture interpatient variability, underscoring its clinical potential.

## Supplementary Material

Supplementary_materials_bbaf317

## Data Availability

Magic is implemented in Python 3.8.19 and is publicly available at https://github.com/cat-moom/Magic, where it will be maintained and regularly updated. The pretrained ViT-B/32 model checkpoint can be accessed at https://drive.weixin.qq.com/s?k=AJEAIQdfAAoUxhXE7r, with the pre-trained wights available at https://dl.fbaipublicfiles.com/deit/deit_base_patch16_224-b5f2ef4d.pth. For scBert, the source code and model are available at the following GitHub repository: https://github.com/TencentAILabHealthcare/scBERT. All data supporting the results of this study are provided in the Supplementary Table S1 of this article.
