# Supplementary material for: Spatial histology and gene-expression representation and generative learning via online self-distillation contrastive learning
Source: Brief Bioinform. 2025 Jul 6;26(4):bbaf317. doi: 10.1093/bib/bbaf317 (PMC12229093; doi:10.1093/bib/bbaf317)
Supplement: Supplementary_materials_bbaf317 [file supplementary_materials_bbaf317.docx]

**Supplementary information of**

**Spatial histology and gene-expression representation and generative learning via online self-distillation contrastive learning**

Qianyi Yan^1,3†^, Xuan Li^2†^, Jiangnan Cui^3^, Jianming Rong^3^, Jingsong Zhang^5^, Pingting Gao^4^, Yaochen Xu^6^, Fufang Qiu^7^, and Chunman Zuo^1,4^*

^1^ School of Life Sciences, Sun Yat-sen University, Guangzhou 510275, China

^2^ College of Computer Science and Technology, Jilin University, Changchun 130012, China

^3^ School of Computer Science and Technology, Donghua University, Shanghai 201620, China

^4^ Shanghai Collaborative Innovation Center of Endoscopy, Endoscopy Center and Endoscopy Research Institute Zhongshan Hospital, Fudan University, Shanghai 200433, China

^5^ Naval Healthcare Information Center, Faculty of Military Health Services, Naval Medical University, Shanghai 200433, China

^6^ Shanghai Institute of Biochemistry and Cell Biology, Center for Excellence in Molecular Cell Science, Chinese Academy of Sciences, Shanghai 200031, China

^7^ Department of Neurosurgery, Huashan Hospital, Shanghai Medical College, Fudan University, National Center for Neruological Disorders, Shanghai Key Laboratory of Bran Function and Restoration and Neural Regeneration, Neurosurgical Institute of Fudan University, Shanghai Clinical Medical Center of Neurosurgery, Shanghai, China

^†^ These authors contributed equally to this work.

* To whom correspondence should be addressed.

Email: zuochm@mail.sysu.edu.cn

**Supplementary Figures**


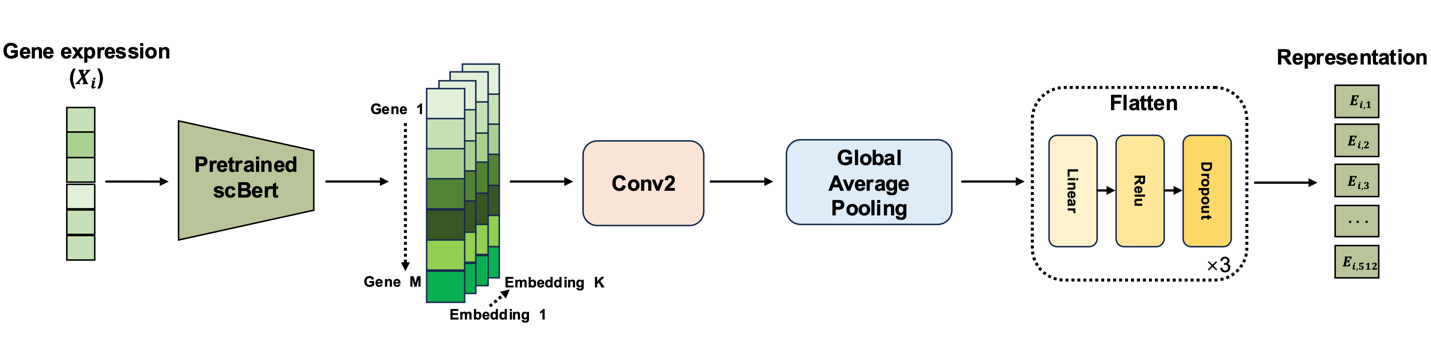


**Figure S1.** Workflow for extracting gene representations from gene expression data using the scBert-based model.


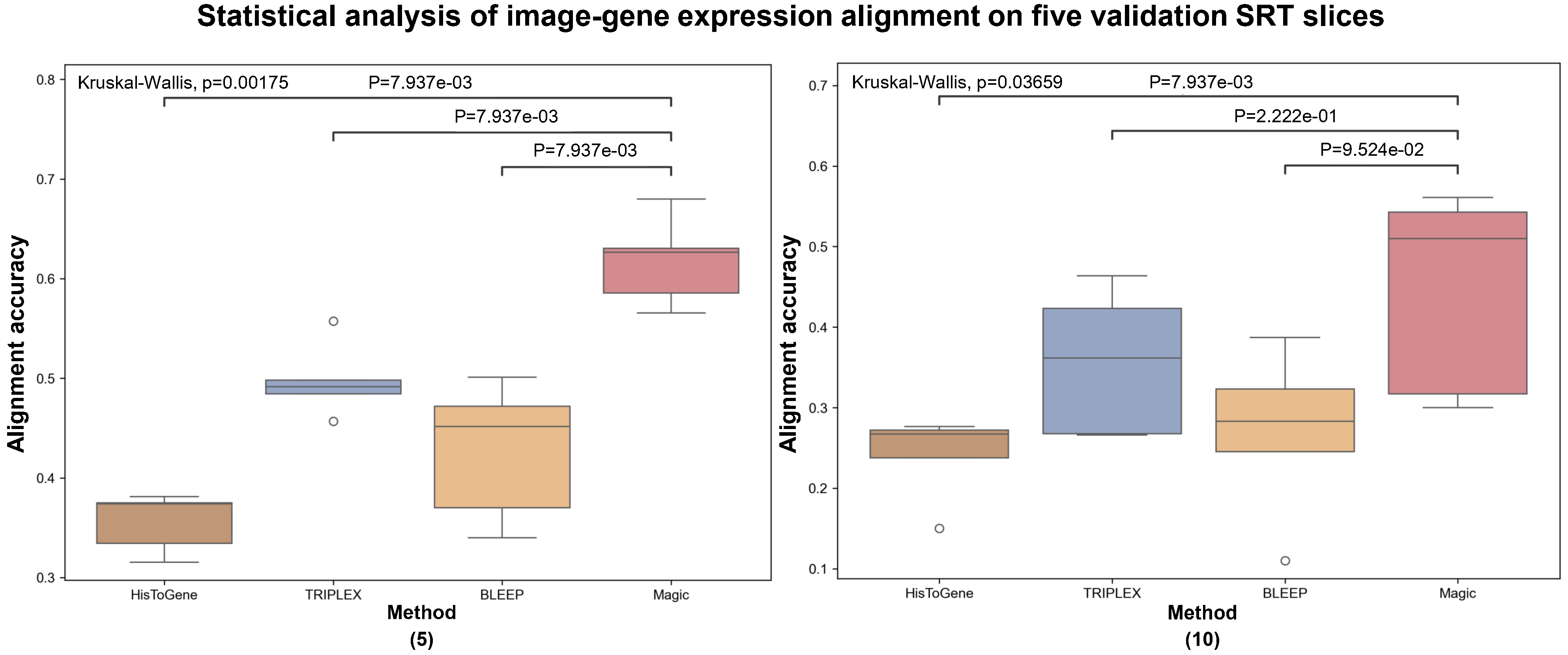


**Figure S2.** Statistical analysis of histology–gene expression alignment using the top 5 and top 10 most similar spots in the low-dimensional feature space across five validation SRT slices.


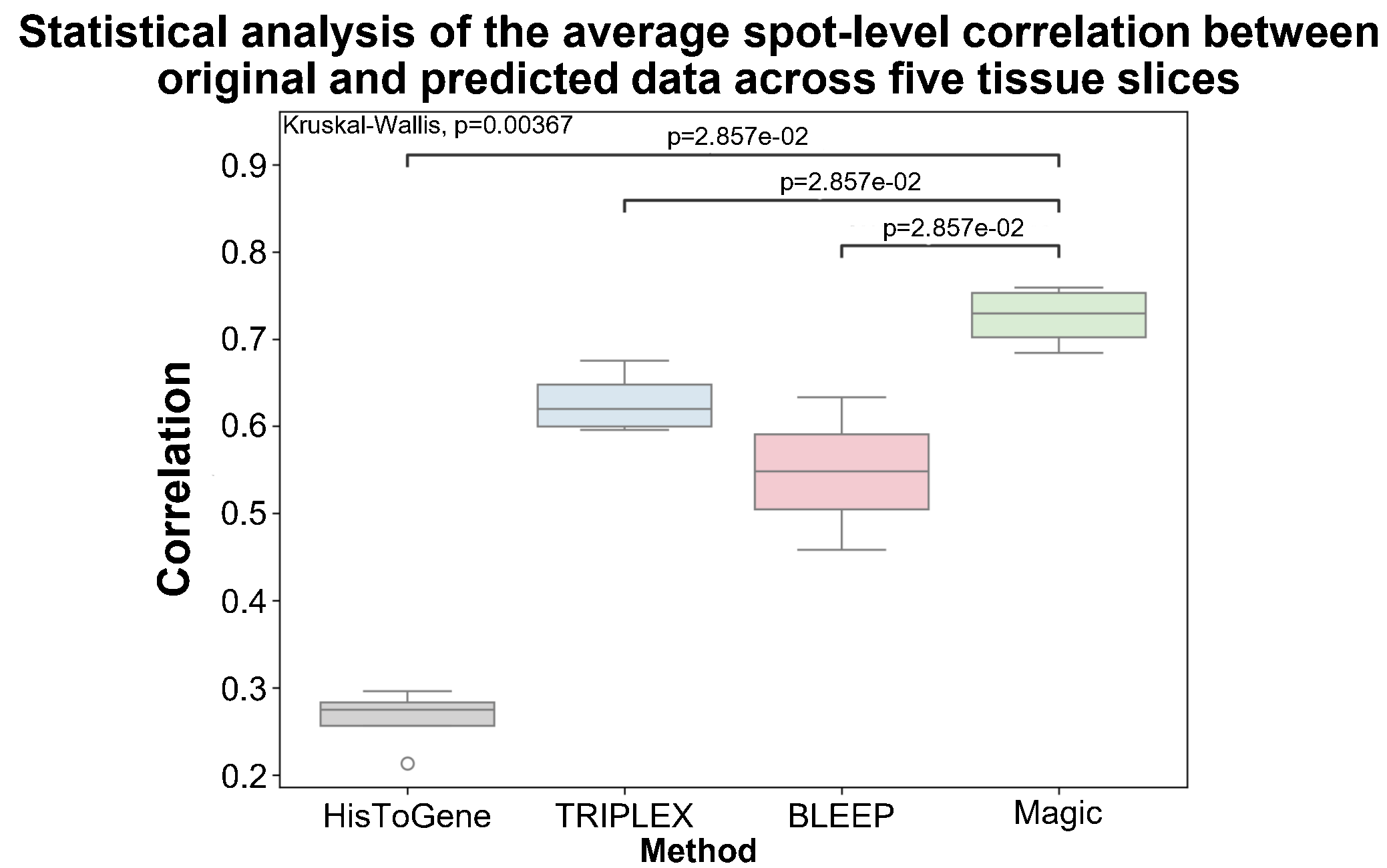


**Figure S3.** Statistical analysis of the average spot-level correlation between original and predicted data across five tissue slices.


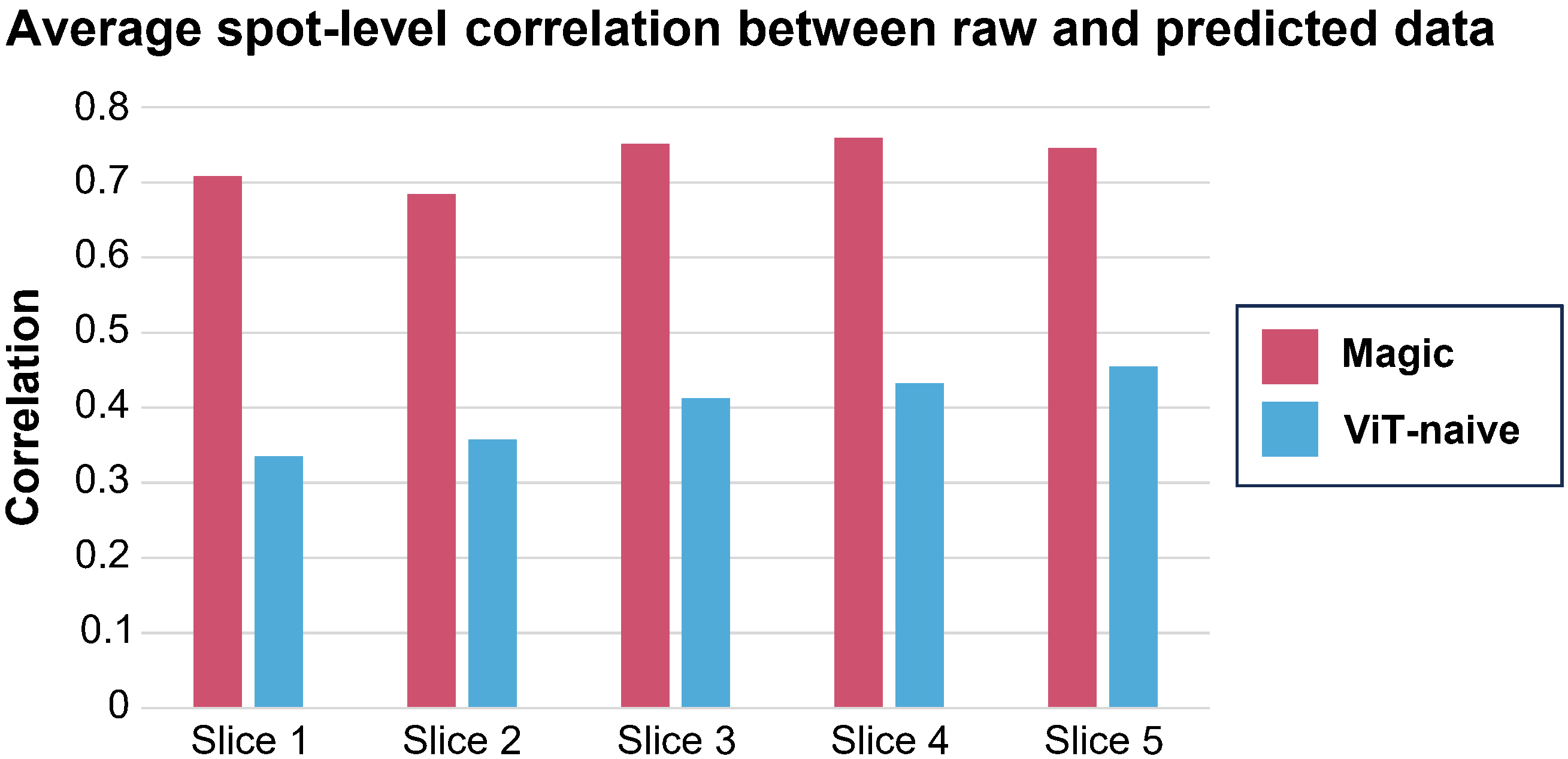


**Figure S4.** Impact of histology-gene expression alignment module on gene expression prediction performance.


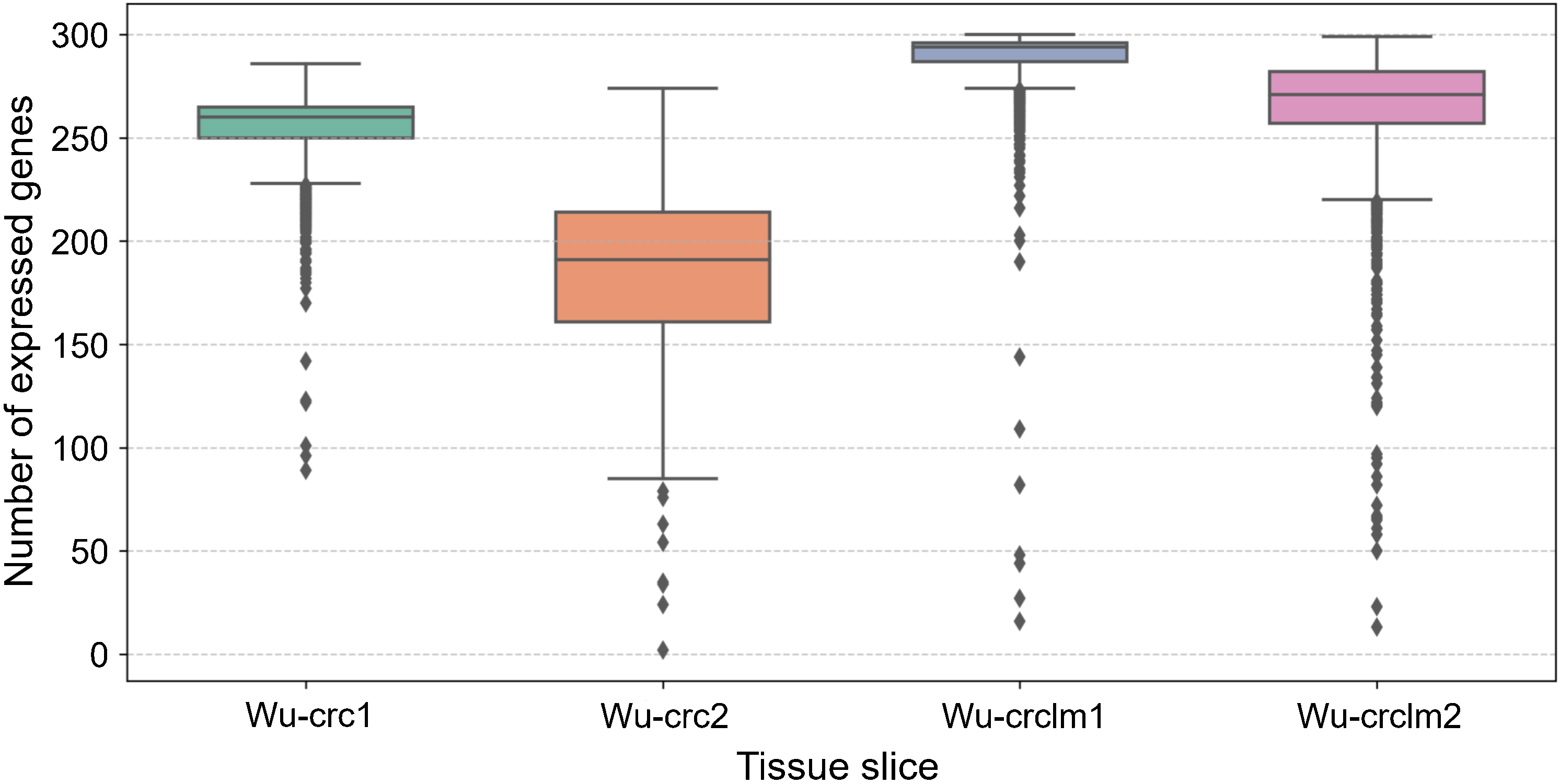


**Figure S5.** Overlap of expressed genes between breast cancer training dataset and colorectal cancer evaluation dataset.


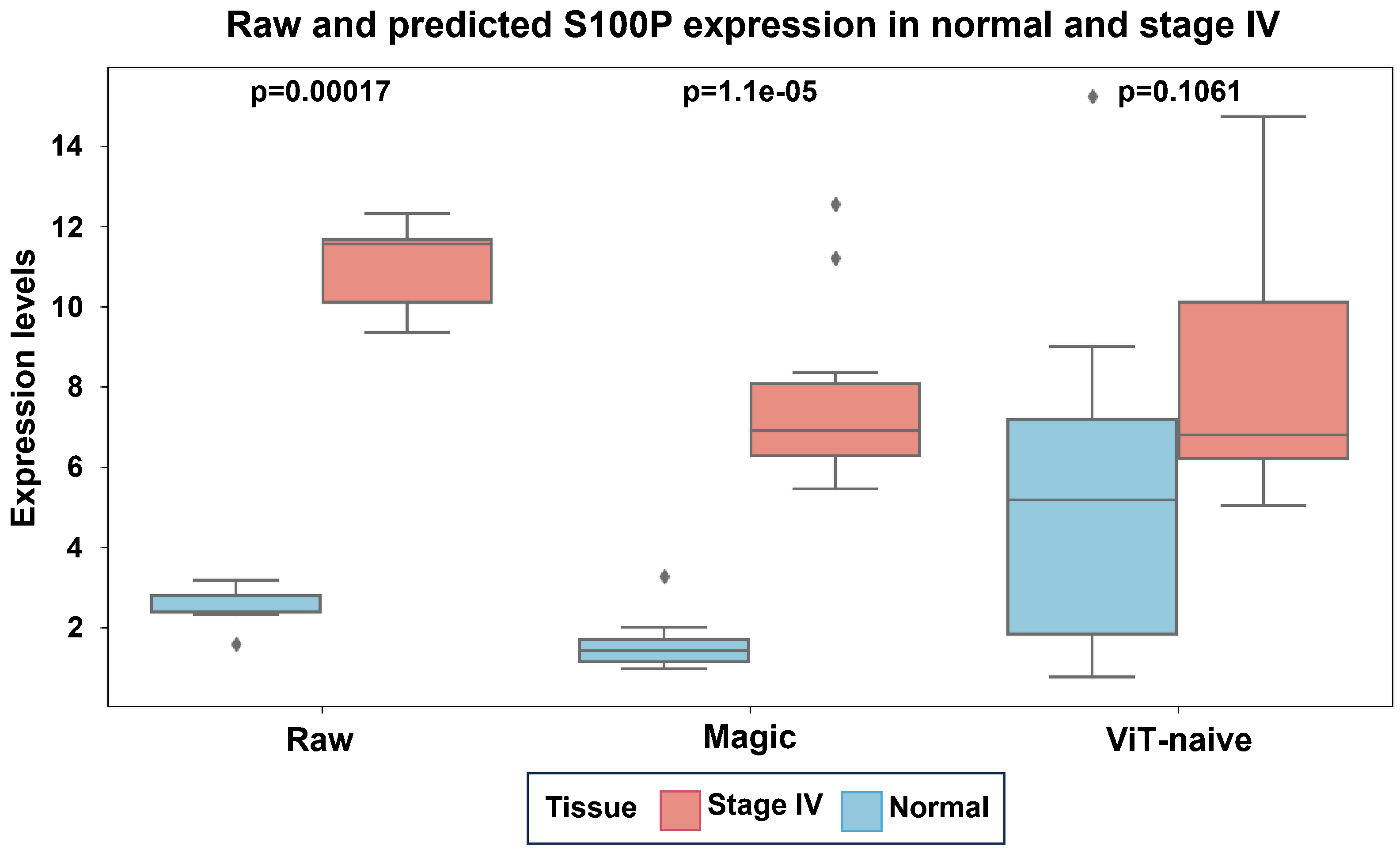


**Figure S6.** Comparison of raw and predicted *S100P* expression levels in normal and stage IV breast cancer tissues.


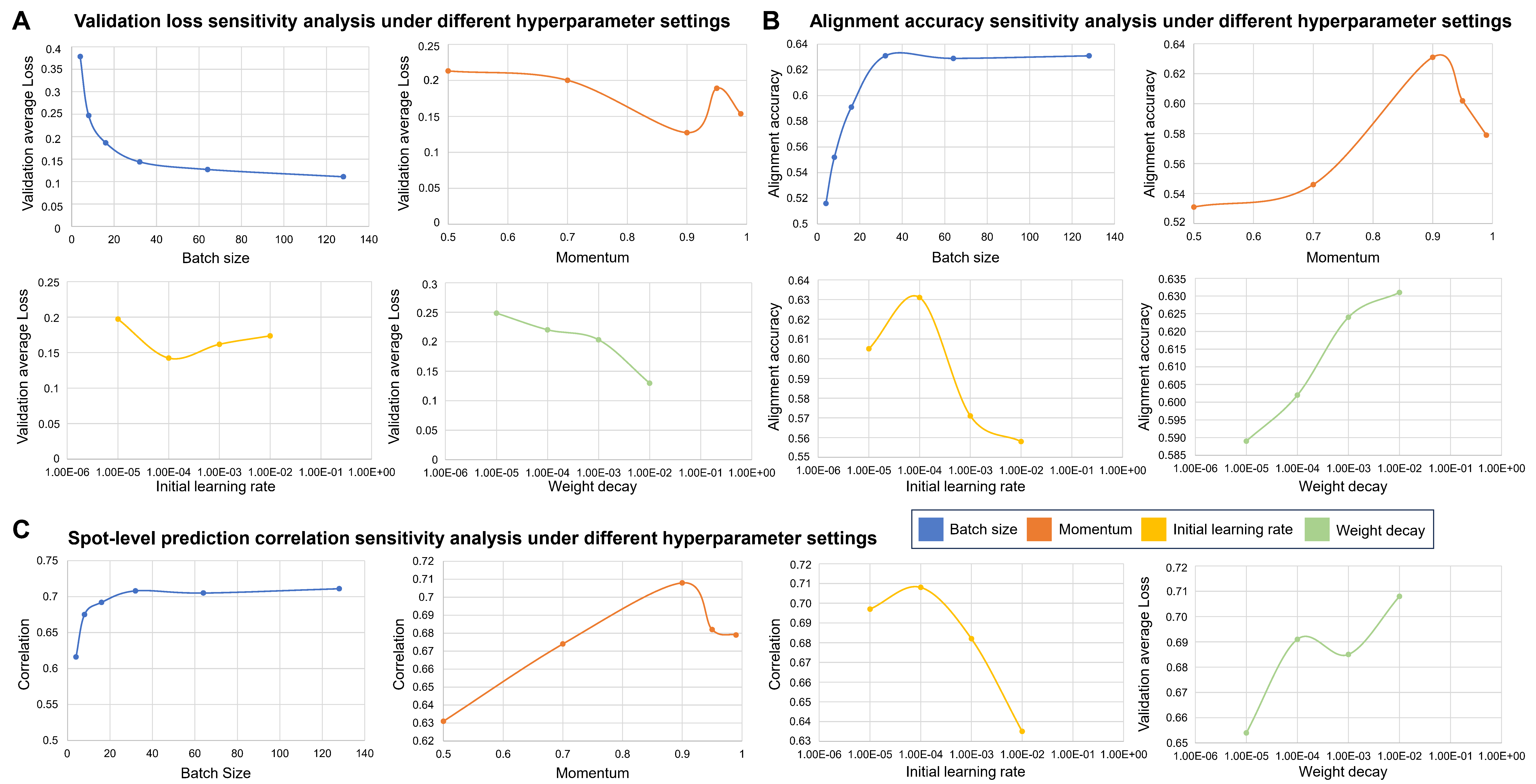


**Figure S7.** Sensitivity analysis of hyperparameters in the Magic model.


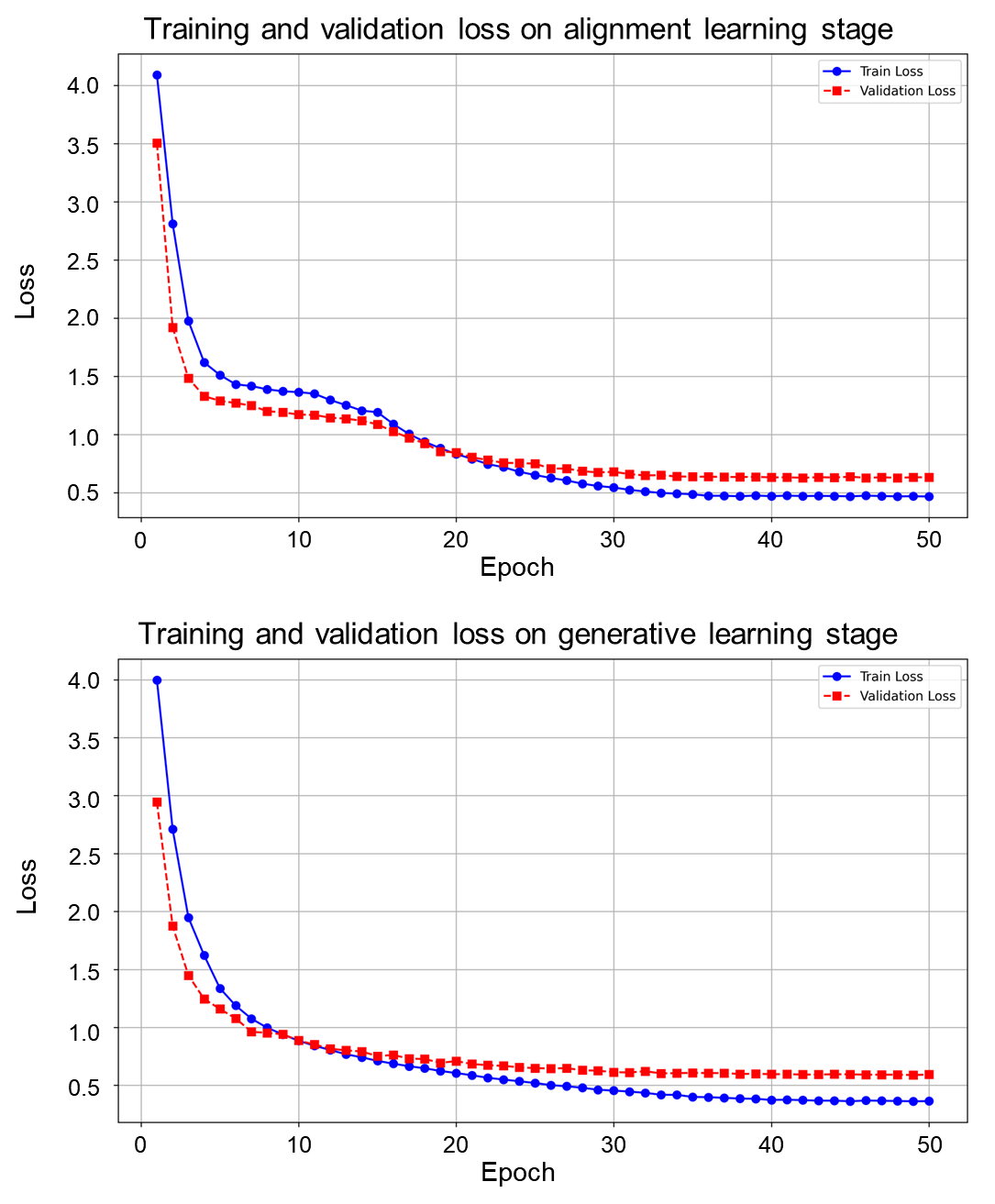


**Figure S8.** Training and validation loss curves for alignment learning stage and generative learning stage of Magic.


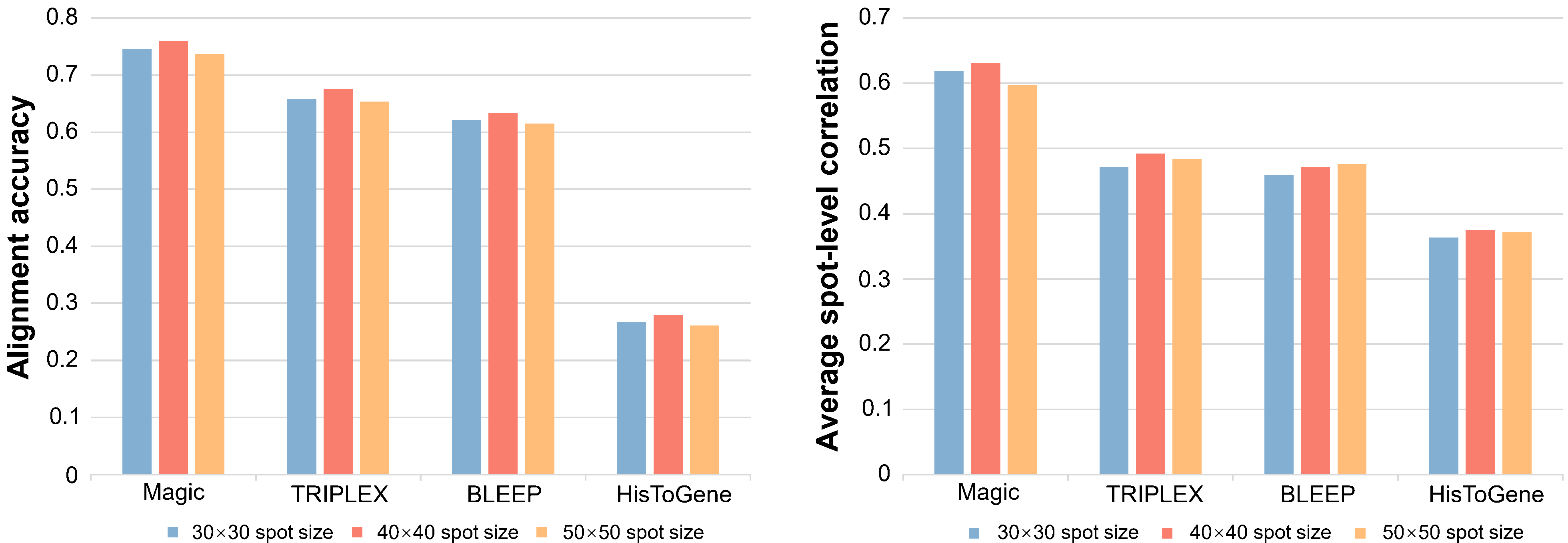


**Figure S9.** Comparative evaluation of model performance across different spot sizes.

**Supplementary Tables**

**Table S1.** Summary of the used dataset.

| **Smaple Name** | **Information** | **Groups** | **Link** |
| --- | --- | --- | --- |
| 1-GSM6433585_092A | TNBC | Training | https://www.ncbi.nlm.nih.gov/geo/download/?acc=GSE210616&format=file |
| 2-GSM6433586_092B | TNBC |  |  |
| 3-GSM6433614_395B | TNBC |  |  |
| 4-GSM6433616_395D | TNBC |  |  |
| 5-GSM6433617_396A | TNBC |  |  |
| 6-GSM6433618_396C | TNBC |  |  |
| 7-GSM6433619_396D | TNBC |  |  |
| 8-GSM6433620_397A | TNBC |  |  |
| 9-GSM6433621_397B | TNBC |  |  |
| 10-GSM6433622_397C | TNBC |  |  |
| 11-GSM6433623_397D | TNBC |  |  |
| 12-GSM6433624_398A | TNBC |  |  |
| 13-GSM6433626_398C | TNBC |  |  |
| 14-GSM6433627_398D | TNBC |  |  |
| 15-GSM6433587_093A | TNBC |  |  |
| 16-GSM6433588_093B | TNBC |  |  |
| 17-GSM6433589_093C | TNBC |  |  |
| 18-GSM6433590_093D | TNBC |  |  |
| 19-GSM6433591_094A | TNBC |  |  |
| 20-GSM6433592_094B | TNBC |  |  |
| 21-GSM6433593_094C | TNBC |  |  |
| 22-GSM6433594_094D | TNBC |  |  |
| 23-GSM6433596_095B | TNBC |  |  |
| 24-GSM6433597_117B | TNBC |  |  |
| 25-GSM6433598_117C | TNBC |  |  |
| 26-GSM6433599_117D | TNBC |  |  |
| 27-GSM6433600_117E | TNBC |  |  |
| 28-GSM6433601_118B | TNBC |  |  |
| 29-GSM6433602_118C | TNBC |  |  |
| 30-GSM6433603_118D | TNBC |  |  |
| 31-GSM6433604_118E | TNBC |  |  |
| 32-GSM6433605_119B | TNBC |  |  |
| 33-GSM6433606_119C | TNBC |  |  |
| 34-GSM6433607_119D | TNBC |  |  |
| 35-GSM6433608_119E | TNBC |  |  |
| 36-GSM6433609_120B | TNBC |  |  |
| 37-GSM6433610_120C | TNBC |  |  |
| 38-GSM6433611_120D | TNBC |  |  |
| 39-GSM6433612_120E | TNBC |  |  |
| 40-GSM6433613_395A | TNBC |  |  |
| 41-BRCA_BlockASection2_10x | Luminal B |  | <http://www.spatialtme.yelab.site/> |
| 42-BRCA_DuctalCarcinomaInSitu_InvasiveCarcinoma_10x_FFPE | IDC |  |  |
| 43-BRCA_Visium_FreshFrozen_WholeTranscriptome_10x | IDC |  |  |
| 44-BRCA_WholeTranscriptomeAnalysis_10x | IDC |  |  |
| 45-BRCA_InvasiveDuctalCarcinoma_StainedWithFluorescent_CD3Antibody_10x | Luminal B |  |  |
| 46-GSM5420753 | Metastatic tumor from BRCA |  |  |
| 47-1142243F | TNBC |  |  |
| 48-1160920F | TNBC |  |  |
| 49-CID4290 | TNBC |  |  |
| 50-CID4465 | TNBC |  |  |
| 51-CID4535 | TNBC |  |  |
| 52-CID44971 | TNBC |  |  |
| 53-GSM6177603_NYU_GIST1 | DCIS |  | https://www.ncbi.nlm.nih.gov/geo/download/?acc=GSE203612&format=file |
| 54-GSM6177603_NYU_BRCA2 | DCIS |  |  |
| 55-GSM6177603_NYU_GIST2 | DCIS |  |  |
| 56-GSM6433615_395C | TNBC |  | https://www.ncbi.nlm.nih.gov/geo/download/?acc=GSE210616&format=file |
| 57-GSM6177603_NYU_BRCA0 | DCIS |  | https://www.ncbi.nlm.nih.gov/geo/download/?acc=GSE203612&format=file |
| 58-GSM6177601_NYU_BRCA1 | DCIS | Validation(Fig3a,e)(Fig2) |  |
| 59-GSM6433595_095A | TNBC | Validation(Fig3a,c,d,e)(Fig2) | https://www.ncbi.nlm.nih.gov/geo/download/?acc=GSE210616&format=file |
| 60-GSM6433625_398B | TNBC | validation(Fig3a,b,e)(Fig2) |  |
| 61-BRCA_BlockASection1_10x | Luminal B |  | <http://www.spatialtme.yelab.site/> |
| 62-ST-colon1 | CRC | Zero-shot learning(Fig4a-e) | https://www.spatialtme.yelab.site/#!/browse/CRC |
| 63-ST-colon2 | CRC | Histology-gene expression alignment accuracy(Fig.4a) |  |
| 64-ST-liver1 | CRC |  |  |
| 65-ST-liver2 | CRC |  |  |
| 66-ST-liver3 | CRC |  |  |
| 67-TCGA-A8-A08J-01A | IDC | *S100P* Prediction (Fig.5c) | https://portal.gdc.cancer.gov/ |
| 68-TCGA-UU-A93S-01A | IDC | *S100P* Prediction (Fig.5c)  Evaluation of zero-shot learning (Fig.5b) |  |
| 69-TCGA-A2-A0SW-01A | IDC |  |  |
| 70-TCGA-PL-A8LX-01A | IDC |  |  |
| 71-TCGA-A8-A08T-01A | IDC |  |  |
| 72-TCGA-A2-A0T2-01A | IDC |  |  |
| 73-TCGA-AO-A0J5-01A | IDC |  |  |
| 74-TCGA-BH-A18J-01A | IDC |  |  |
| 75-TCGA-B6-A0IB-01A | IDC |  |  |
| 76-TCGA-A2-A0CS-01A | IDC |  |  |
| 77-TCGA-E9-A1N5-11A | IDC |  |  |
| 78-TCGA-BH-A0HA-11A | IDC |  |  |
| 79-TCGA-BH-A1FG-11B | IDC |  |  |
| 80-TCGA-E2-A1IG-11A | IDC |  |  |
| 81-TCGA-BH-A1FR-11B | IDC |  |  |
| 82-TCGA-E9-A1ND-11A | IDC |  |  |
| 83-TCGA-BH-A18P-11A | IDC |  |  |
| 84-TCGA-BH-A1FE-11B | IDC |  |  |
| 85-TCGA-BH-A203-11A | IDC |  |  |
| 86-TCGA-BH-A0DD-11A | IDC |  |  |
| 87-TCGA-AC-A3W5-01A | IDC |  |  |
| 88-TCGA-GM-A2DN-01A | IDC | Evaluation of zero-shot learning (Fig.5b) |  |
| 89-TCGA-B6-A0RS-01A | IDC |  |  |
| 90-TCGA-E2-A15C-01A | IDC |  |  |
| 91-TCGA-A8-A09I-01A | IDC |  |  |

**Table S2.** Comparison of image-gene expression alignment accuracy across different methods in five validation slices on the breast cancer dataset.

| **Method** | **Slice 1** | **Slice 2** | **Slice 3** | **Slice 4** | **Slice 5** | **Mean** |
| --- | --- | --- | --- | --- | --- | --- |
| HisToGene | 0.374 | 0.3155 | 0.3343 | 0.3813 | 0.375 | 0.36 |
| TRIPLEX | 0.5574 | 0.498 | 0.45689 | 0.4844 | 0.4916 | 0.50 |
| BLEEP | 0.4517 | 0.37 | 0.5012 | 0.34 | 0.472 | 0.43 |
| Magic | 0.68 | 0.5854 | 0.6265 | 0.5657 | 0.6305 | 0.62 |

**Table S3.** Comparison of image-gene expression alignment accuracy across different methods in five experiments on the colorectal cancer dataset.

| **Method** | **Sample 1** | **Sample 2** | **Sample 3** | **Sample 4** | **Sample 5** | **Mean** |
| --- | --- | --- | --- | --- | --- | --- |
| HisToGene | 0.242 | 0.18 | 0.152 | 0.182 | 0.198 | 0.19 |
| TRIPLEX | 0.354 | 0.184 | 0.284 | 0.298 | 0.216 | 0.27 |
| BLEEP | 0.362 | 0.306 | 0.33 | 0.386 | 0.316 | 0.34 |
| Magic | 0.506 | 0.398 | 0.418 | 0.514 | 0.424 | 0.45 |

**Table S4.** Comparison of method in training time, memory usage, and inference speed. Experiments were conducted on a server with a 16-core AMD Ryzen 9 5950X CPU (64GB RAM) and two NVIDIA RTX 3090 GPUs (24GB each).

| **Model** | | **Model training time** | **Memory consumption** | **Inference time** |
| --- | --- | --- | --- | --- |
| HisToGene | | ~1 hours | ~12 GB | ~80 milliseconds |
| BLEEP | | ~1.5 hours | ~16 GB | ~80 milliseconds |
| Magic | Image-Expression Alignment Stage | ~2 hours | ~18 GB | ~180 milliseconds |
|  | Expression Generation Stage | ~0.5 hours | ~18 GB | ~80 milliseconds |
| TRIPLEX | | ~2.5 hours | ~22 GB | ~300 milliseconds |

**Table S5.** Hyperparameter settings for the Magic model.

| **Hyperparameters** | **Parameter Introduction** | **Setting value** |
| --- | --- | --- |
| batch_size | The number of samples in each batch during training. | 32 |
| Init_Epoch | The initial epoch number at the beginning of training. | 0 |
| Epoch | The maximum number of iterations during training. | 100 |
| Init_lr | The initial learning rate, controlling the step size for parameter updates. | 1e-4 |
| Min_lr | The minimum learning rate during training. | Init_lr * 0.01 |
| momentum | The decay rate of the first-moment estimate in the optimizer, affecting the speed of gradient updates. | 0.9 |
| weight_decay | The L2 regularization term, used to mitigate overfitting. | 1e-2 |
| lr_decay_type | The type of learning rate decay, determining how the learning rate changes during training. | 'cos' |

**Table S6.** Definitions of key symbols in the Magic model.

| **Symbol** | **Definition** |
| --- | --- |
| $Conv2(\cdot)$ | 2D convolutional layer, used for extracting local features. |
| $GAP(\cdot)$ | Global Average Pooling layer, used for dimensionality reduction and extracting global features. |
| $ReLU(\cdot)$ | Nonlinear activation function. |
| $Dropout(\cdot)$ | Randomly drops neuron outputs to prevent overfitting. |
| $LayerNorm(\cdot)$ | Layer Normalization, used to stabilize training and accelerate convergence. |
| $Concat(\cdot)$ | Feature concatenation operation, used to combine multiple features into a single representation. |
| $MultiHead(\cdot)$ | Multi-Head Attention, used to capture information from different subspaces. |
| $FFN(\cdot)$ | Feed-Forward Network, used for feature transformation and nonlinear mapping. |
| $CrossAttention(\cdot)$ | Cross-attention mechanism, used to model interactions between different features. |
| $Softmax(\cdot)$ | Normalization function that converts inputs into a probability distribution. |
| $Attention(\cdot)$ | Self-attention mechanism, used to compute weighted representations of input features. |
| $tanh(\cdot)$ | Hyperbolic tangent activation function, used to scale values to the range (-1, 1). |
| $sim(\cdot)$ | Cosine similarity function, used to measure the closeness between two feature vectors. |
